# Systematic Review of Mindfulness-Based Interventions in Child-Adolescent Population: A Developmental Perspective

**DOI:** 10.3390/ejihpe12080085

**Published:** 2022-08-22

**Authors:** Bárbara Porter, Cristian Oyanadel, Fabiola Sáez-Delgado, Ana Andaur, Wenceslao Peñate

**Affiliations:** 1 Departamento de Psicología, Facultad de Ciencias Sociales, Universidad de Concepción, 4030000 Concepción, Chile; 2 Centro de Investigación en Educación y Desarrollo y Facultad de Educación, Departamento Fundamentos de la Pedagogía, Universidad Católica de la Santísima Concepción, 4030000 Concepción, Chile; 3 Escuela de Psicología, Pontificia Universidad Católica de Chile, 8331150 Santiago, Chile; 4 Departamento de Psicología Clínica, Psicobiología y Metodología, Facultad de Psicología, Campus de Guajara, Universidad de La Laguna, 38200 Santa Cruz de Tenerife, Spain

**Keywords:** mindfulness, childhood, adolescence, youth mental health, developmental psychology, socioemotional competencies, executive functions

## Abstract

Human development implies deep changes in cognitive, attentional, emotional, and behavioral skills. Therefore, Mindfulness-Based Interventions (MBIs) should be adapted in terms of dose, frequency, kind of exercises, assessment methods, and expected effects regarding the abilities and limitations of each developmental period. The present review seeks to describe and compare MBIs characteristics, assessment methods, and effects in youth between 3 and 18 years old considering four developmental periods. A systematic review was carried out including experimental primary studies published during the last five years. Results show that the frequency of the sessions and program duration varies widely. Differences were observed in instructors’ training and in assessment strategies. Discrepancies were observed regarding the effects of MBIs both within and between periods in cognitive, socio-emotional, symptoms, and mindfulness variables. Consistency was observed in prosocial behaviors for preschoolers, and in emotional and behavioral problems and hyperactivity in ages between preschool and early adolescence. Nevertheless, it was impossible to compare most results and determine consistency or discrepancy due to the lack of studies. Regarding mindfulness, it is defined and assessed in different ways in each period. Orientations are suggested to move from a compartmentalized view of isolated MBIs, towards an integrative perspective that allows tracing developmental trajectories for mindfulness and other key cognitive and socioemotional skills for children and adolescents.

## 1. Introduction

Common mental disorders are a relevant cause of morbidity in youth [1]. Around 20% of the world’s children and adolescents have a mental health condition; depression and anxiety are the most common mental health disorders in the child and adolescent population, causing significant developmental effects [2]. Lack of access to adequate treatment is the common rule [3]. Failure to address child and adolescent mental health problems has important consequences, since these problems hinder the achievement of basic aspects of development [4] and have lifelong effects. They also generate a high rate of disability [5], since The DALYs (Disability-Adjusted Life Years) rate is especially high for 10 to 19 years old. Due to the high demand related to child and adolescent mental health issues, and the scarcity of resources destinated to address this problem [6,7], the need of moving towards a universal and preventive intervention model, instead of an individual and reactive intervention model arises [8].

Over the past decades, Mindfulness-based interventions (MBIs) have gained popularity, particularly in the domain of psychological well-being and symptom reduction [9]. This has led to the development of a plethora of child and adolescent universal and clinical interventions based on mindfulness [10]. Paying attention, in a particular way, with a purpose, in the present moment, and without judgment, is considered the classic definition of mindfulness in western psychology [11,12]. It has been defined as a two-component construct: (a) self-regulation of attention, which is maintained in the immediate experience, and (b) orientation to one’s own present experience characterized by curiosity, openness, and acceptance [13]. The off-center perspective of observing experiences without judgment [14] is a skill that can be trained and can have therapeutic effects [15].

MBI’s effects have been assessed abundantly in the past decade through experimental and quasi-experimental designs, showing small to medium size effects in the reduction of depression symptoms [16,17,18,19,20], anxiety [21,22,23,24,25,26], and stress [27,28,29,30]. In child and adolescent population, MBIs have been related to symptom reduction, increased wellbeing [1,10,14,15,31,32,33,34,35,36], attention regulation [37,38], emotional regulation [39,40] and behavioral regulation [41,42,43]. Despite some encouraging results, a lack of understanding about the practices within MBIs that can foster specific skills or reduce determined symptoms prevails. MBIs usually mix different practices, content, and dosage [44]. In addition, some MBIs are applied to a wide range of populations, including participants of different ages, clinical conditions, cultures, and socioeconomic backgrounds. Consequently, the evidence is not clear regarding the most appropriate type of interventions for each group, or the variables that may be more feasible to modify in each context. Socioeconomic factor has been addressed recently in a systematic review (SR) that regards MBI results in low-income schools [45]. Nevertheless, the developmental perspective has not been analyzed yet as a critical factor that can determine the success or failure of an MBI.

It is well known that human beings go through different developmental periods, in which they contemplate the acquisition and strengthening of various skills [46], although there is a controversy regarding the real consideration of developmental stages [47]. Contemporary developmental psychology has an orientation that holds an integrated relational model of human life, that synthesizes biological-through-physical ecological influences in a way that considers the complexity of human development. Additionally, the field has shown a growing appreciation of the importance of the cultural and historical influences on the quality and trajectory of human development across the course of life [48]. Therefore, it is not easy to separate life span in precise groups of specific ages, because there is always an interplay of different factors that will influence child development, increasing intragroup differentiation. Additionally, different processes develop at different timing and trends; this means that core aspects of development (e.g., language, executive functions, empathy, among many other intertwined processes) can be traced in developmental trajectories. However, there is some agreement in the literature about the general grouping: infants (or early childhood), childhood (sometimes called middle childhood), and adolescence (sometimes differentiating between early, middle, and late adolescence). Differences arise in the cutting age of each group [49,50]. For the present work we consider that, although this perspective has limitations, it provides a relevant frame for choosing effective interventions for a specific population. A panoramic developmental perspective is relevant because it considers the particularities, potentialities, and limitations of each developmental stage. Based on this view, the type of intervention and exercises, dose of practice, intervention goals, and assessment strategies can be chosen more precisely and effectively. Therefore, we will consider both views: a developmental period classification and a longitudinal view of specific developmental trajectories. For the developmental period classification, we will consider Roberts and DelVecchio [50] and Ferguson [49] on personality metanalytical research work. To classify the primary studies based on different developmental stages, we used the reported average age of each sample. Considering the average age of the sample of each primary study, we classified them into 4 groups: (1) Preschool period (3 to 6 years average sample age); (2) Middle Childhood (7 to 10.9 average sample age); (3) Early Adolescence (11 to 13.9 average sample age); (4) Middle Adolescence (14 to 18 average sample age). This classification gives some clear parameters that help with the following analysis. However, we understand the overlap between these groups and the intertwined developmental trajectories of different aspects of human development, which will be also considered and explained in the result analysis.

It is important to know what has been done in the research aimed at understanding the effects of MBIs in childhood and adolescence. However, even though some reviews have systematized the available evidence, some questions have not yet been answered, such as the similarities or differences between the MBIs applied at each developmental period, or the consistency or discrepancy regarding the effects of the MBIs both within and between stages.

In the context of the present study, a search for Systematic Reviews and Meta Reviews published in the last 10 years was conducted. 11 SRs and MR were found, which were analyzed based on their objectives, screened databases, search date, and reported limitations (Table 1). As shown in Table 1, they are a good attempt to summarize the effects and characteristics of MBIs in children and adolescents. Nevertheless, the following limitations are observed: (1) Only consider primary studies with a population belonging to some specific stages of development [35,51,52]; (2) Only consider a population with a specific socioeconomic condition [45]; (3) Consider effects of MBI on specific symptoms [1]; (4) Include only qualitative studies [36]; (5) Interventions include non-mindfulness practices (yoga, body image, SEL, Cognitive Therapy [10,51]; (6) The focus of the review is to determine the degree to which interventions align to MBI standards [53]; (7) Include primary studies conducted past 5 years [14,54,55].

Deep changes are produced in our cognitive, attentional, emotional, and behavioral skills during the human lifespan [46]; therefore, we must consider that mindfulness practice can produce different effects depending on the developmental stage, especially during childhood and adolescence. We need to have a complete scope of the main effects of this practice during all developmental stages to choose wisely. No Systematic Review has been conducted to address this objective. The present article seeks to elucidate the effectiveness of MBIs in childhood and adolescence between 3 and 18 years old, considering a developmental perspective. The purpose is to highlight the differences and similarities of interventions, assessment methods, and main effects of MBI in each developmental stage. To meet this objective a systematic review was carried out. Primary studies with experimental designs published during the last 5 years in various digital databases were included. Main effects were reported within and between 4 groups: (1) Preschool period (3 to 6 years average sample age); (2) Middle Childhood (7 to 10.9 average sample age); (3) Early Adolescence (11 to 13.9 average sample age); (4) Middle Adolescence (14 to 18 average sample age). The general objective was to describe and analyze empirical research with experimental designs that have implemented and assessed the effects of MBIs on children and adolescents with typical development considering four different stages of development (early childhood, childhood, early adolescence, and adolescence). Specifically, three objectives were established:

Objective 1. Describe Mindfulness-based interventions for children and adolescents considering 4 different developmental periods according to the following aspects: (a) Sample mean age, (b) Sample size, (c) Reported name of interventions, (d) Intervention location, (e) Duration (Weeks of intervention), (f) Total numbers of sessions, (g) Sessions frequency, (h) Session duration, (i) Person who delivers the intervention, (j) Type of exercises, (k) Intervention modality (face to face; online synchronous; online asynchronous).

Objective 2: Analyze the types of evaluation strategy (observation, self-report scale, performance task, computerized task, physiological measures) used to measure the effectiveness of the intervention, according to each developmental period.

Objective 3: Analyze the effectiveness of the interventions over dependent variables considered according to each developmental period.

## 2. Method

### 2.1. Sources/Literature Research

A systematic review of studies published in WoS, Scopus, PubMed, and EBSCO (Psych articles, and Behavioral Sciences Collection) were conducted. A total of 1587 initial references were considered. Primary studies published in English and Spanish were included, from the year of the last available published review that considered all childhood and adolescence periods (Carsley, Khoury, et al. 2018) till May 2021. The search strategy was a combination of the following terms: Title: (mindfulness) AND TOPIC: (“child” OR “adolescence” OR “adolescents” OR “teenagers” OR “teens” OR “youth” OR “school”). The search was refined considering Publishing years (2017 TO 2021) AND type of document: (ARTICLE) AND Language: (ENGLISH OR SPANISH) AND categories: (PSYCHOLOGY CLINICAL OR PSYCHOLOGY MULTIDISCIPLINARY OR PSYCHOLOGY DEVELOPMENTAL OR PSYCHOLOGY EDUCATIONAL OR PSYCHOLOGY APPLIED OR PSYCHOLOGY OR PSYCHOLOGY EXPERIMENTAL).

The references of primary articles were inspected. After removing duplicates, the titles and abstracts of the remaining studies were screened. Finally, full-text articles were assessed to check if they met inclusion criteria. The five authors verified the retrieval process. Finally, 27 primary studies were considered in the present analysis.

### 2.2. Selection Criteria

Inclusion criteria were: (1) The intervention must include classic mindfulness exercises (mindful breathing, body-scan, mindful walking, mindful movement); (2) Sample age between 3 and 18 years old; (3) Nonclinical population; (4) MBI carried out in school context; (5) Experimental or Randomized Controlled Trial (RCT) design; (6) Outcome must include at least one psychological variable.

### 2.3. Exclusion Criteria

Studies were excluded if: (1) Interventions were shorter than 1 session of 40 min; (2) MBI was conducted in other contexts (not school); (3) If randomization was done in clusters, within 3 or fewer groups; (4) Intervention included the participation of parents or other adults (not as instructors).

## 3. Results

**Figure 1:**
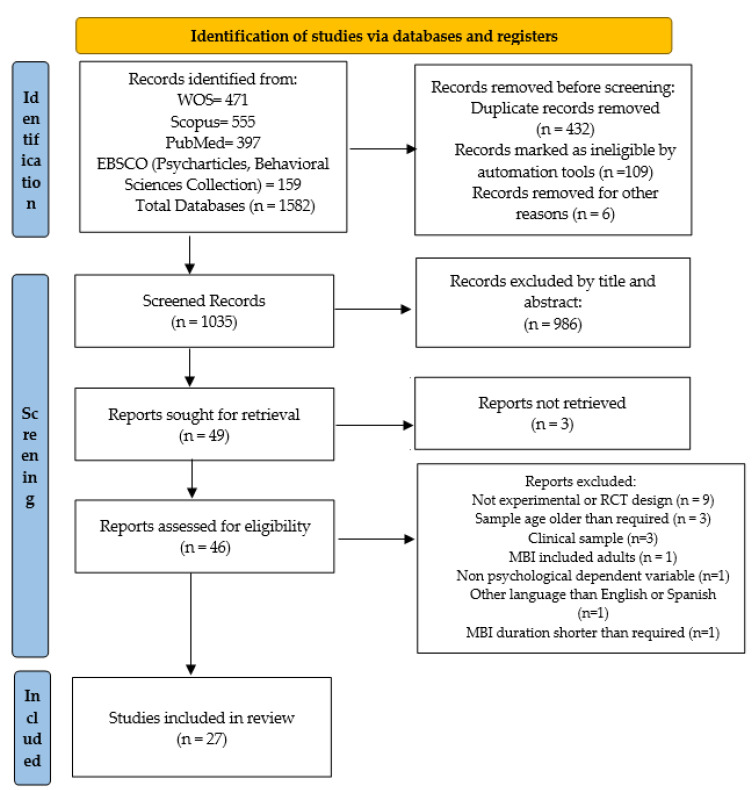
PRISMA flow diagram of the search and selection process. From: Page MJ, McKenzie JE, Bossuyt PM, Boutron I, Hoffmann TC, Mulrow CD, et al. The PRISMA 2020 statement: an updated guideline for reporting systematic reviews [56].

Preferred Reporting Items for Systematic Reviews and Meta-Analyses (PRISMA) guidelines were used to examine reporting in a systematic way. A total of 1582 records were retrieved in the literature search. After removing duplicates and eliminating non-peer-reviewed studies, a total of 1035 articles were left. They were screened by title and abstract, checking the study method and sample. Doubtful cases were checked and solved by 2 independent judges. A total of 161 studies were sought for retrieval and were checked by screening the full text. Finally, 46 studies were assessed for eligibility, based on inclusion and exclusion criteria. A total of 19 articles were excluded because they were not experimental studies (9 studies), the sample average age was older than required (3), the sample had a clinical diagnosis (3), and were published in a different language than required (1), the MBI included adults (1), included only non-psychological dependent variables (1), or the implemented intervention was shorter than required (1). A total of 27 articles were considered eligible for inclusion through this combined search strategy (Figure 1). The methodological quality of these reviews was examined using the Cochrane Guidelines. Studies included in this review meet minimum quality standards set by Cochrane Guidelines.

All 27 studies were coded with an ID (Table 2) and analyzed later in base of an extraction matrix that included information related to each one of the objectives.

To facilitate the mention of each of the 27 primary studies, an ID number was assigned to each one, following the references correlative numbering, by which they will be referred to from now on.

In order to meet the first objective, MBIs for children and adolescents were analyzed considering four different developmental periods described before (early childhood, childhood, early adolescence, and adolescence) according to the following aspects: (a) Sample mean age, (b) Sample size, (c) Reported name of interventions, (d) Intervention location, (e) Duration: Weeks of intervention (f) Total numbers of sessions, (g) Sessions frequency, (h) Session duration, (i) Person who delivers the intervention, (j) Type of exercises, (k) Intervention modality (face to face; online synchronous; online asynchronous) (Table 3).

As shown in Table 3 a plethora of different interventions were reported. Each program has a specific duration, number of sessions, frequency, and session duration. The shorter intervention was done in 4 weeks, and the longest in 24 weeks. In addition, the frequency of the sessions varies widely (1 per week, to 5 per week) within and between periods. Related to the duration of each session, differences can be observed between periods: Preschool period shows the shorter sessions (20–40 min), meanwhile early and middle adolescence show the longer ones (40–90 min).

In the same way, a variety of instructors deliver the interventions, such as trained graduate research assistants, instructors with basic proficiency in mindfulness, trained authors, clinical psychologists, or class tutors. It is reported that some of them completed MBSR training, while others received short-term training before delivering the program.

Another important finding was related to the type of exercises and intervention modality. A greater variety of exercises was observed during early developmental periods, especially in the informal practices, which were restricted only to the classic practices in older populations. Since the authors do not usually report the guidelines of the performed practices, a deep comparison between exercises between periods was not possible. It would be interesting to know whether the same reported exercise (e.g., mindful breathing) is performed in a similar or different way between developmental periods.

Related to the intervention modality, all MBIs were delivered face-to-face. Just one of them was done online, asynchronously, with an adolescent population [79].

The second objective of this SR was to analyze the types of evaluation strategy (observation, self-report scale, performance task, computerized task, physiological measures) and the instruments used to assess the effectiveness of the intervention, according to developmental periods. To meet this objective, all the information regarding assessment strategy and instruments was shown in Table 4.

As we can see in Table 4, in the early periods a variety of assessment strategies were used. In fact, observational strategies were used only in the preschool stage. In the same way, physiological measures are still scarce. On the other hand, a great abundance of self-report scales was observed especially in middle and late adolescence, to the detriment of other assessment strategies. Regarding self-report tests, it was observed that the same tests were applied in different developmental stages. In some cases, authors applied the child (MAAS-C) or adolescent version (MAAS-A), due to the age of their target population. Nevertheless, since authors usually do not report the questions of the tests they apply, a more detailed analysis is needed to determine whether the language of the questions was adapted for each period in the other self-report tests (e.g., FFMQ was applied in periods 2 and 4).

The third and last objective was to analyze the effectiveness of the interventions over the dependent variables according to each developmental period. To meet this goal, a result analysis was conducted, considering all main variables described in each primary research (Table 5, Table 6, Table 7, Table 8 and Table 9). Main dependent variables that showed a significant statistical change after intervention (*p* < 0.5) were marked with *, and the ones that did not show change were marked with +. Moreover, we analyzed these results to determine whether MBIs’ effects over a specific variable were consistent within and between developmental periods. Consistency meant that two or more studies show similar effects (e.g.: both studies show a significant statistical change in the same variable) discrepancy meant that though one or more studies show a significant change in a specific variable, others do not. This consistency or discrepancy occurred within the same period (e.g., two studies with preschool children show similar or different results over a specific dependent variable) or between periods (e.g., the variable shows a significant change in a study with preschoolers but another study, with early adolescents do not show a significant change in that same variable).

All dependent variables were reported, and grouping them into five categories, within each of the four developmental periods: (1) Cognitive (Table 5), (2) Socioemotional (Table 6), (3) Symptoms (Table 7), (4) Mindfulness (Table 8) and (5) Visual-motor skills and physiological measures (Table 9). Moreover, in the cognitive and socioemotional categories, we considered sub-categories considering critical developmental trajectories. In cognitive category we considered (1) Executive Functions [84,85]; (2) Attentional Skills [86]. In the Socioemotional category, we based our analysis on CASEL Model [87] considering (1) Self-management skills; (2) Social Awareness; (3) Relationship skills; (4) Intergroup Relationships and; (6) Classroom climate. Wellbeing, and others, where the last two sub-categories considered in the analysis of socioemotional variables. Symptoms were classified considering Achenbach’s terms of internalizing and externalizing [88,89,90,91].

**Table 3:** Mindfulness-based interventions for children and adolescents’ characteristics for each developmental period.

Stage	ID	Mean Age	N	Name	Place	Duration	N° of Sessions	Frecuency	Session Duration	Instructor	Type of Excercises/Intervention Modality
1	[62,66,67,68,70,73,78]	3 to 6 years and 5 months	21 to 218	Mindful Schools Program, Mini Mind, Calmer Choice, Open Mind Korea, not reported	Daycare, Preeschool, Child development center, school	6 to 8 weeks	12 to 32 sessions	2 till 5 sessions per week	20 to 40 min each session	Certified Instructor, local trained teacher, primary researcher, graduate students with previous training.	Informal practice: Mindful listening, mindful eating, watching clouds, glitter jar. Formal practice: mindful breathing, body scan, mindful eating, mindful movement, mindful emotion awareness, loving kindness practices. All face to face.
2	[58,60,61,65,76]	8.5 to 10.25	101 to 400	Pause, breathe, smile, Call to Care Israel, Gaia	School	8 to 24 weeks	8 to 24 sessions	1 per week	45 to 60 min	Trained author, trained research assistants, Instructors with basic proficiency in mindfulness.	Informal practice: Mindful listening, mindful eating, meditation bubble, imagining own safe peaceful place. Formal practice: mindful breathing, body scan, yoga, mindful walking, mindful emotion awareness, loving kindness practices. All face to face.
3	[63,64,69,71,72,75,80,81,82]	11.58 to 13.5 years	22 to 3519	SEA program, Learning to Breathe, Kamalay Curriculumb., Eline Snel program	School, boarding school	6 weeks to 5 months	6 to 32 sessions	1 per month to 4 per week	40 to 90 min	Trained clinical psychologist, Tutors of class group, trained instructors.	Informal practice: Mindful listening, mindful eating, mindful task awareness, mindful relationship awareness. Formal practice: mindful breathing, body scan, yoga, mindful walking, mindful emotion awareness, mindful thoughts awareness, loving kindness practices. All face to face.
4	[57,59,74,77,79,83]	14.5 to 17 years	30 to 1349	MBSR (adapted), Learning to Breathe, Meditacion Fluir, Mindfulness Psychoeducation, Mindfulness-based well-being course.	School, afterschool	4 to 10 weeks	8 to 12 sessions	1 or 2 per week	45 to 90 min	MBSR trained instructor, author, trained schoolteachers.	Informal practice: mindful listening, attention to 5 senses, mindful behavior awareness. Formal practice: mindful breathing, mindful body awareness, body scan, mindful awareness of emotions, mindful awareness of thoughts, loving kindness practices. Face to face and online asynchronous.

**Table 4:** Types of evaluation strategy used for each developmental period.

Stage	Observational Assessment	Self-Report Scale	Performance Task	Computerized Task	Physiologic Measure
1	(1) Classroom Assessment Scoring System (in CLASS) (2) Child Observation Mindfulness Measure (C-OMM) (3) Non-participant naturalistic observations based on Roche Olivar’s prosocial behavior assessment scheme	(1) Teacher version of the Strengths and Difficulties Questionnaire (SDQ) (2) Minnesota executive function scale (MEFS) (3) Theory of mind scale (4) Children’s Behavior Questionnaire (CBQ; Very Short Form) (5) Child Behavior Rating Scale (CBRS) (6) Emotion Regulation Checklist (ERC) (7) Korean Personality Rating Scale for Children (KPRC) (8) Modified Professional Behavioral Questionnaire (Mod-PBQ) (9) Rating form for teachers to assess executive functions was grounded in the literature review of EF skills for preschoolers	(1) Head-toes-knees-shoulders (2) Peg tapping (3) Dimensional Change Card Sort Task (4) Sally-Ann task (5) The unexpected-content task (6) Deceptive object task (7) Visual perception tasks (8) Hiding game	(1) Flanker Inhibitory Control and Attention Test (2) Go/No Go	Not assessed
2	Not assessed	(1) Stirling Children’s Wellbeing Scale (SCWBS) (2) Mindful Attention Awareness Scale adapted for Children (MAAS-C) (3) Spence Children’s Anxiety Scale (SCAS) (4) FFMQ (5) Readiness for Social Contact measure (6) BAffective Prejudice scale (7) BStereotyping measure (8) Teacher’s Report Form (TRF/6-18; Achenbach System of Empirically Based Assessment ASEBA)	(1) Beery–Buktenica Developmental Test of Visual-Motor Integration (VMI) (2) Conjunctive Visual Search Task	(1) Computerized Continuous Performance Task (CPT)	Not assessed
3	Not assessed	(1) Self-Regulatory Inventory (ASRI) (2) Rosenberg Self-esteem Scale (3) Ryff’s Psychological Well-being scale (4) Child Adolescent Mindfulness Measure (CAMM) (5) Mindfulness scale for school scope (6) Perceived Stress Scale (PSS) (7) Positive and Negative Affect Schedule for Children ( PANAS-C) (8) Response to Stress Questionnaire (9) Mindful Attention Awareness Scale (MAAS—C) (10) Test Bull-S form A (11) Mi vida en el instituto (12) Escala de Distancia Social (13) Barratt Impulsiveness (14) Strengths and Difficulties Questionnaire (SDQ) (15) Short Mood and Feelings Questionnaire (SMFQ) (16) State-Trait Anxiety Inventory for Children (STAIC) (17) Difficulties in Emotion Regulation Scale (DERS) (18) The Resilience scale (RS14) (19) Beck Depression Inventory (BDI) (20) Youth Self-Report (YSR) (21) Behavior Rating Inventory of Executive Function Self Report (BRIEF-SR) [92] (22) A single-item measure of perceived stress level developed by the program developer (23) Ruminative Responses Scale (RRS)	(1) Face match performance during fMRI (2) Working Memory Index (WMI) (3) Digit span subtest from the WISC-IV (4) NEPSY-II (5) Trail Making test and Verbal Fluency sub test from D-KEFS	Not assessed	(1) fMRI
4	Not assessed	(1) Perceived Stress Scale (PSS-10) (2) Optimism (EQ-i, YV) (3) Emotional Quotient Inventory (EQ-i, YV) (4) Mindfulness (FFMQ) (5) Screen for Child Anxiety and Related Emotional Disorders (SCARED.) (6) Child Acceptance and Mindfulness Measure (CAMM) (7) Escala Atribucional de Motivación de Logro (EAML) (8) Cuestionario de Estrategias y Motivación para el Aprendizaje (MSLQ) (9) Mindfulness Attention Awareness Scale (MAAS) (10) The Values in Action Inventory of Strengths for Youth/VIA-Youth (11) Two-factor Revised Beck Depression Inventory Finnish version (R-BDI;) (12) Finnish School Burn-out Inventory (13) Short-form World Health Organization Quality of Life (WHOQoL-BREF (14) Satisfaction with Life Scale (SWLS) (15) Basic Nordic Sleep Questionnaire (16) Happiness item from UN’s World Happiness Report (17) Self-Compassion Scale-Short Form (SCS-SF) (18) Difficulties in Emotion Regulation Scale (DERS) (19) Patient Health Questionnaire (PHQ-8) (20) Generalized Anxiety Disorder Scale (GAD-7) (21) Rumination subscale of the Rumination and Reflection Questionnaire (RRQ) (22) Stress was measured with two subscales of the Adolescent Stress Questionnaire (ASQ) (23) The Adolescent Sleep-Wake Scale (ASWS) (24) Social Connectedness Scale Revised (SCC-R) (25) Mind Wandering Questionnaire (MWQ) (26) Implicit Theories of Intelligence Scale for Children (IT) (27) Substance Initiation Index (28) Young Adult Alcohol Problems Screening Test (YAAPST)	Not assessed	(1) Automated Operation Span Task (AOSPAN) (2) Computerized version of the Stroop Task (3) Modified version of the Balloon Analogue Risk Task (BART) (4) Modified Emotional Faces N-back Task (EFN-back)	Not assessed

**Table 5:** MBI effects over cognitive dependent variables (significant statistical change and consistency analysis) *.

Period	Skills	Variables	Discrepancy or Consistency of Results ID (Period)
1	Executive Functions	Task Orientation +	No significant changes
		Working Memory *+	Discrepancy between periods: 67 (1) *, 59 (4) *//81 (3) +
		Executive Functions *+	Discrepancy within period: 68 (1) *,70 (1) *, 81 (3) *//67 (1) +
		Self-Regulation (inhibitory behavioral control) +	No significant changes
	Attentional Skills	Sustained Attention *+	Discrepancy between periods 65 (2) *//62 (1) +
		Inhibition +	No significant changes
		Shifting +	No significant changes
2	Attentional Skills	Sustained Attention *+	Discrepancy between periods 65 (2) *//62 (1) +
		Selective Attention *	One study cannot compare: 64 (2) *
3	Executive Functions	Rote Memory *	One study, cannot compare: 81 (3) *
		Working Memory *+	Discrepancy between periods: 67 (1) *, 59 (4) *//81 (3) +
		Response Inhibition *	One study, cannot compare: 81 (3) *
		Cognitive Processing *	One study, cannot compare: 81 (3) *
		Cognitive Flexibility *	One study, cannot compare: 81 (3) *
		Self-monitoring *	One study, cannot compare: 80 (3) *
	Others	Verbal Fluency *	One study, cannot compare: 81 (3) *
4	Executive Functions	Working Memory *+	Discrepancy between periods: 67 (1) *, 59 (4) *//81 (3) +
	Attentional Skills	Inhibitory Control attention +	No significant changes
		Attention Regulation +	No significant changes
	Others	Academic Achievement *	One study, cannot compare: 68 (4)

Note: Variables column: * Significant statistical change post-intervention; + No significant change; *+ Discrepant changes within or between periods (2 or more studies). Discrepancy/Consistency column: ID (period) * Significant statistical change; //ID (period)+ non-significant change post intervention.

**Table 6:** MBI effects over socioemotional dependent variables (significant statistical change and consistency analysis) *.

Period	Skills	Variables	Discrepancy or Consistency of Results ID (Period)
1	Self Managment Skills	Emotion regulation *+	Discrepancy between periods 83 (4) *//78 (1) +, 72(3) +, 80 (3) +
	Social Awareness	Emotional Perspective Taking *	One study, cannot compare: 73 (1) *
		Cognitive Perspective Taking *	One study, cannot compare: 73(1) *
		Visual Perspective Taking +	No significant changes
		Theory of Mind +	No significant changes
	Relationship Skills	Teacher Interaction +	No significant changes
		Peer Interaction +	No significant changes
		Prosocial behavior **	Consistency within period: 66 (1) *, 73 (1) *, 78 (1) *
	Others	Resilience **	Consistency between periods: 78 (1) *, 75 (3) *
2	Relationship Skills	Readiness for social contact *	One study, cannot compare: 61 (2)
	Intergroup Relationships	Affective Prejudice *	One study, cannot compare: 61 (2)
		Stereotyping *	One study, cannot compare: 61 (2)
	Wellbeing	General Wellbeing *	One study, cannot compare: 58 (2)
		Subjective Wellbeing *	One study, cannot compare: 58 (2)
		Psychological Wellbeing *+	Discrepancy between periods 63 (3) *//58 (2) +
3	Self Managment Skills	Self-regulation (sum, short, long term) *	One study, cannot compare
		Emotion Regulation *+	Discrepancy between periods 83 (4) *//78 (1) +, 72(3) +, 80 (3) +
		Emotional Control +	No significant changes
		Primary Coping +	No significant changes
		Socio-emotional functioning *	One study, cannot compare: 75 (3) * Significant change only for girls
	Intergroup Relationships	Social Distancing +	No significant changes
		Bullying +	No significant changes
	Classroom Climate	Social Climate (positive and negative) *	One study, cannot compare 82 (3) *
	Wellbeing	Self-esteem *	One study, cannot compare: 63 (3) *
		Psychological Wellbeing *+	Discrepancy between periods: 63 (3) *//58 (2) +
	Others	Resilience **	Consistency between periods: 78 (1) *, 75 (3) *
4	Self Managment Skills	Emotion regulation *+	Discrepancy between periods 83 (4) *//78 (1) +, 72(3) +, 80 (3) +
		Risk Taking +	No significant changes
		Achievement Motivation *	One study, cannot compare: 74 (4) *
		Intrinsic, Extrinsic, Learning Motivation +	No significant changes
		Task Appreciation +	No significant changes
	Intergroup relationships	Social Connectedness *	One study, cannot compare: 83 (4) * change only group adequate practice
	Wellbeing	Psychological Quality of Life *	One study, cannot compare: 79 (4) *
	Others	Character Strengths *	One study, cannot compare: 77 (4) *
		Optimism +	No significant changes

Note: Variables column: * Significant statistical change post-intervention; + No significant change; ** Consistent significant change (2 or more studies); *+ Discrepant changes within or between periods (2 or more studies). Discrepancy/Consistency column: ID (period) * Significant change; //ID (period)+ non-significant change.

**Table 7:** MBI effects over symptoms (significant statistical change and consistency analysis) *.

Period	Kind of Symptoms	Variables	Discrepancy or Consistency of Results ID (Period)
1	Internalizing Symptoms	Emotional problems (SDQ subscale) **	Consistency between periods: 70 (1) *, 75 (3) *
	Externalizing Symptoms	Hyperactivity **	Consistency within and between periods: 66 (1) *, 70 (1) *, 76 (2) *, 75 (3) *
		Conduct Problems **	Consistency between periods: 70 (1) *, 75 (3) *
2	Internalizing Symptoms	Anxiety Symptoms *+	Discrepancy within and between periods: 60 (2) *, 80 (3) *, 79 (4) *//72 (3) +, 59 (4) +, 83 (4) +
		Depressive Symptoms *+	Discrepancy within and between periods: 76 (2) *, 75 (3) *, 79 (4) *//72 (3) +, 83 (4) +
		Somatic Complaints *	Cannot compare, only one study: 76 (2)
	Externalizing Symptoms	Hyperactivity **	Consistency within and between periods: 66 (1) *, 70 (1) *, 76 (2) *, 75 (3) *
		Inattention *	Cannot compare, only one study: 76 (2)
		Rule Breaking *	Cannot compare, only one study: 76 (2)
		Aggressive Behavior *	Cannot compare, only one study: 76 (2)
3	Internalizing Symptoms	Perceived Stress *+	Discrepancy within and between periods: 69 (3) *, 57 (4) *//59 (4) +
		Depressive Symptoms *+	Discrepancy within and between periods: 76 (2) *, 75 (3) *, 79 (4) *//72 (3) +, 83 (4) +
		Anxiety Symptoms *+	Discrepancy within and between periods: 60 (2) *, 80 (3) *, 79 (4) *//72 (3) +, 59 (4) +, 83 (4) +
		Difficulties in Emotion Regulation +	No significant changes.
		Rumination *+	Discrepancy between periods: 80 (3) *, 83 (4) *, (study 83 reported higher rumination in experimental group post intervention)
		Emotional problems (SDQ subscale) **	Consistency between periods: 70 (1) *, 75 (3) *
	Extrernalizing symptoms	Hyperactivity **	Consistency within and between periods: 66 (1) *, 70 (1) *, 76 (2) *, 75 (3) *
		Conduct Problems **	Consistency between periods: 70 (1) *, 75 (3) *
		Negative Affect *	Cannot compare, only one study: 69 (3)
		Impulsivity *	Cannot compare, only one study: 82 (3)
4	Internalizing Symptoms	Perceived Stress	Discrepancy within and between periods: 69 (3) *, 57 (4) *//59 (4) +
		Anxiety Symptoms *+	Discrepancy within and between periods: 60 (2) *, 80 (3) *, 79 (4) *//72 (3) +, 59 (4) +, 83 (4) +
		Depressive Symptoms *+	Discrepancy within and between periods: 76 (2) *, 75 (3) *, 79 (4) *//72 (3) +, 83 (4) +
		Sleep problems +	No significant changes.
	Externalizing Symptoms	Substance Abuse +	No significant changes.
		Negative substance Abuse consequences +	No significant changes.

Note: Variables column: * Significant statistical change post-intervention; + No significant change; ** Consistent significant change (2 or more studies); *+ Discrepant changes within or between periods (2 or more studies). Discrepancy/Consistency column: ID (period) * Significant statistical change; //ID (period) + non-significant change post intervention.

**Table 8:** MBI effects over mindfulness variables (significant statistical change and consistency analysis) *.

Period	Variables	Discrepancy or Consistency of Results ID (Period)
1	Orientation to experience *	Cannot compare, only one study: 61 (1) *
	Self-Regulated Attention +	No significant changes.
2	General Mindfulness (FFMQ) **	Consistency between periods: 60 (2) *, 57 (4) *
	Observing **	Consistency between periods: 60 (2) *, 57 (4) *
	Describing **	Consistency between periods: 60 (2) *, 57 (4) *
	Acting with awareness **	Consistency between periods: 60 (2) *, 57 (4) *
	Non-judging **	Consistency between periods: 60 (2) *, 57 (4) *
	Non-reacting **	Consistency between periods: 60 (2) *, 57 (4) *
	Trait Mindfulness (MAAS-C) *+	Discrepancy between periods: 58 (2) *, 77 (4) *//71 (3) +
3	Kinesthetic Mindfulness *	Cannot compare, only one study: 64 (3) *
	External Mindfulness +	No significant changes.
	Internal Mindfulness *	Cannot compare, only one study: 64 (3) *
	Mindfulness (CAMM) *+	Discrepancy between periods: 63 (3) *//59 (4) +, 79 (4) +, 83 (4) +
4	General Mindfulness (FFMQ) **	Consistency between periods: 60 (2) *, 57 (4) *
	Observing **	Consistency between periods: 60 (2) *, 57 (4) *
	Describing **	Consistency between periods: 60 (2) *, 57 (4) *
	Acting with awareness **	Consistency between periods: 60 (2) *, 57 (4) *
	Non-judging **	Consistency between periods: 60 (2) *, 57 (4) *
	Trait Mindfulness (MAAS-C) *+	Consistency between periods: 60 (2) *, 57 (4) *
	Mindfulness (CAMM) *+	Discrepancy between periods: 63 (3) *//59 (4) +, 79 (4) +, 83 (4) +
	Mind Wandering *	Cannot compare, only one study: 83 (4) *

Note: Variables column: * Significant statistical change post-intervention; + No significant change; ** Consistent significant change (2 or more studies); *+ Discrepant changes within or between periods (2 or more studies). Discrepancy/Consistency column: ID (period) * Significant statistical change; //ID (period) + non-significant change post intervention.

**Table 9:** MBI effects over physiologic and viso-motor variables (significant statistical change and consistency analysis) *.

Period	Kind of Variables	Variables	Discrepancies/Consistency of Results
2	Visual-motor Skills	Visual Perception *	Cannot compare, only one study: 60 (2) *
		Motor Accuracy *	Cannot compare, only one study: 60 (2) *
3	Physiologic Measures	Amygdala Reactivity *	Cannot compare, only one study: 69 (3) *

Note: Variables column: * Significant statistical change post-intervention. Discrepancy/Consistency column: ID (period) * Significant statistical change.

## 4. Discussion

The first objective of this review was to describe MBIs for children and adolescents considering the different stages of development (pre-school period, middle childhood, early adolescence, and adolescence) according to the age, duration, number, duration, and frequency of sessions, person who delivers the intervention, type of exercises and intervention modality (face to face; online synchronous; online asynchronous).

Related to the total duration of the program, number, and frequency of sessions, great variability was observed, not showing consistency within each developmental stage. This is important because the total duration and the number of sessions or frequency are not determined by specific developmental needs. The only parameter that can be related to developmental issues is the duration of each session, which shows a consistent difference between periods. These parameters should be assessed considering the most effective formula for each developmental period, in the same way that MBSR has been considered the golden standard for the adult population [11,12]. Since the ability to sustain attention is a skill that develops over time, it is important to consider the evolutionary aspect in the design of MBI sessions. Subjecting a child to an excessively prolonged practice can produce the opposite desired effect, causing rejection, resistance, or anxiety.

In the same way, there is no global standard about the minimum training and practice required to be able to deliver mindfulness programs for children and teens, as was reported by Emerson [53], which can be an obstacle to assessing the effects of MBIs, due to the interference of instructor proficiency.

Related to the type of exercises, a variety of practices were observed at early ages, especially in the informal practices, restricting to the most classic ones in the adolescent population. This self-imposed restriction could hinder the adherence of adolescents to MBI, diminishing the variety of mindfulness practices that can be shared with them. Although it is known that a mindfulness intervention must have certain basic exercises such as breathing awareness, body scan, attention to movement, and mindful walking [12], it would be interesting not to give up the variety of practices that can be done with adolescents, to address their motivation and commitment with the practice. On the other hand, although there is a difference in the methodology used (use of concrete material in the early stages vs. classic exercises in adolescents), there is no greater variation in the complexity of the skills to be developed. Nevertheless, a deeper analysis is needed, to compare the guidelines of the performed practices.

Related to the intervention modality, almost all were delivered face to face. Just one program (for adolescents) was delivered online [79]. Considering the growing need for effective online interventions due to the pandemic context, its necessary to develop this trend. Online interventions present opportunities and limitations. A significant opportunity is the possibility to overcome geographical limitations and ease access to a population that, otherwise, would not be able to take part in this kind of interventions (vulnerable or rural sectors). Therefore, online interventions can be universal and cheaper, since the logistical cost of rooms and transfers are avoided. Concrete material is important to facilitate mindfulness practice in the early stages and could be a limitation of online interventions. However, this problem can be solved easily, by using material that is accessible at home (recycling) or sending the material via courier to the children’s homes. Among the limitations, the access to the internet is relevant, and the risk that the practice of mindfulness becomes an additional online activity, such as math or science classes, represents one more activity to do and an eventual burden for students.

The second objective of this RS was to analyze the different assessment strategies (observation, self-report scale, performance task, computerized task, physiological measures) and the instruments used to assess the effectiveness of the intervention, according to stages of development. Although it is obvious that observational assessments were used with preschoolers due to the impossibility of applying self-report scales, the almost exclusive preference for self-report scales in later stages must be questioned. Although they have adequate psychometric indicators, must be complemented with other evaluation strategies. As stated by Thorndike and Thorndike-Christ [93], paper and pencil tests cannot appropriately assess many instructional objectives and cognitive functions. More direct or authentic assessment is required for some of them. For example, it can be recommended to use systematic observation for some constructs (such as peer relationship skills). In this context, it is important to choose the assessment methods according to the complexity of the variable we are evaluating. Thus, can be necessary to complement self-report scales with other strategies such as observation guidelines, performance tasks, computerized tasks, or physiological measures, in addition to self-report scales applied to other informants (teachers, parents). As quoted by Shrout and Rodgers [94], the generalizability and veracity of results can be assessed if we can conceptually replicate a result, that may involve a similar but not identical intervention, alternate outcome measures, or samples from a distinctly different population. Therefore, a variety of assessment strategies is needed.

The third objective was to analyze the effectiveness of the interventions over the dependent variables considered in the studies according to each developmental period. 5 categories were considered: (1) Cognitive, (2) Socioemotional, (3) Symptoms, (4) Mindfulness, and (5) Visual-motor skills and physiological measures. Cognitive, socioemotional and symptoms categories included sub-categories related to core skills developmental trajectories. Regarding the cognitive variables, it is noteworthy that the executive functions were evaluated in 3 of the 4 stages (stages 1, 3 and 4) presenting in stage 2 (7 to 11 years) only the evaluation of attentional skills. Although attention skills could be closely related to executive functions, they would not be part of them. Some studies consider selective attention as a precursor to executive functions [86]. On the other hand, attentional variables are only evaluated in stages 1, 2, and 4. This discontinuity of variables that are evaluated between the stages could be related to a lack of developmental perspective when selecting the cognitive variables to be developed through an MBI. It would be interesting to incorporate an integrated view of development, operationalizing both the executive functions and the attentional variables according to each stage (e.g., how they manifest, develop, and can be assessed) to have a continuous and non-compartmentalized perspective of their development.

On the other hand, there were significant changes in certain executive functions in stages 1 and 3, but not in stage 4. It will be necessary to analyze whether this is due to variables associated with the intervention (duration, frequency, type of exercises), the instructor’s proficiency, or developmental particularities of adolescence, which would make it more complex to develop these skills within this period.

The assessment of executive functions is an example of the probable role that plays the assessment method on the effects of MBIs. As shown in Table 5, executive functions were assessed in stage 1. In one primary study, the assessment method of EF was a self-report scale completed by the teachers [67]. No significant results were obtained pre-post intervention. On the other hand, a primary study [68] that assessed executive functions used performance tasks (more direct measures) showing significant changes pre-post intervention. In this case, the assessment method could have affected the results. Therefore, it is important to consider the complexity of factors involved when attributing certain effects to an MBI. It will be necessary to consider not only the characteristics of the intervention but also the suitability of the assessment methods and the characteristics of each developmental period.

Considering the natural skills development within each period is important too. For example, in stage 1, the theory of mind is included as a dependent variable [68]. Theory of mind (TOM) is children’s understanding of persons’ mental states, which according to Wellman and Liu [95] can be sequentially developed from almost 3 to 5 years old. In the referred primary study [68] no significant changes were found in TOM between the experimental and control groups. One possible explanation for this could be that the children in the control group also developed this ability due to normal developmental effects. We can ask then, is it relevant to try to promote this ability during this developmental period through the MBI, given that there is an effect associated with natural socio-cognitive development? On the other hand, variability in TOM development can be seen between individuals. Therefore, is it worth promoting this ability only in individuals who may present a lower development than expected for their stage of development? This kind of question should be done to consider the interplay between the MBI effects and the natural developmental trajectories of different skills along a lifespan.

Regarding MBIs effects over symptoms, a significant change was observed in most of them through the different stages, which would indicate that the MBI effectively helps relieve discomfort during childhood and adolescence. Nevertheless, the effectiveness in reducing symptoms seems not to be the same along different stages. In earlier stages (1 and 2) the effects of MBIs in symptom reduction seem to stand out over adolescence, in which the relief seems to happen just in some of them. It would be necessary to ponder the reason for that. One hypothesis may be that adolescents, being more aware of their discomfort, are more likely to answer self-report scales in a self-critical way. Once again, the assessment methods and the particularities of each developmental stage can be playing a role.

A significant effect of MBIs could be seen in physiological measures and visual-motor skills. However, only 2 primary studies include them, with a population belonging to stage 2, middle childhood, [60] and 3, early adolescence [69]. It would be interesting to include these kinds of variables and assessment methods in stages 1 and 4.

Regarding Mindfulness, a central variable to affirm the internal validity of the studies, important differences in the definition, operationalization, and assessment was observed between developmental periods. Each primary study defined and assessed mindfulness differently. For example, in stage 1 mindfulness was considered as an orientation to experience, a skill evaluated by an observer [62] while in stage 2 [60] mindfulness was defined based on the classic facets of the 5FMQ, considering General Mindfulness, Observing, Describing, Acting with awareness, Non-judging, Non-reacting [96]. It is complex to compare MBIs effects if mindfulness is defined and evaluated differently according to each developmental period. One way to solve this would be to define mindfulness as a unified construct, considering Wilson [97] latent variable model. Considering a developmental perspective, mindfulness, as a latent variable, could progressively increase in complexity according to each developmental stage. Therefore, mindfulness could be assessed through different observed items, according to the level of complexity expected for each developmental stage, maintaining construct coherence and consistency along a lifespan.

Another important issue is the consistency of MBIs effects over certain variables within or between developmental periods. Consistent results can be glimpsed in a few variables, such as prosocial behavior in preschoolers (stage 1); emotional and conduct problems in preschoolers (stage 1) and early adolescents (stage 3); hyperactivity in ages between preschoolers and early adolescents (stages 1, 2 and 3), and mindfulness (general and five factors of FFMQ, for periods 2 and 4). Discrepant results within or between developmental stages were observed for cognitive and socioemotional variables, symptoms, and mindfulness. Most variables were assessed only in one study, so could not be compared. These gaps should be addressed in future studies, to be able to demonstrate more robustly the consistency of MBI effects, or, on the other hand, to understand which factors may be at the base of discrepant effects considering if this occurs within the same period of development, or between different stages.

Based on the results of this study and the analysis carried out, the following practical guidelines can be proposed: (1) Change from a compartmentalized perspective to an integrated view of skills development during childhood and adolescence in the planning and delivery of MBIs. Based on this holistic view, the skills that are expected to be developed through the MBI can be defined, considering strengths, limitations, and expected standards at each developmental period. As the spectrum of cognitive, emotional, social, and motor skills available to children and adolescents becomes more complex, participants can be challenged to a greater degree. (2) Design MBIs based on the evidence by testing and considering the best formula for each development period in terms of program duration, frequency, and types of exercises. In relation to the types of exercises, it is important that the variability is not only in terms of the kind of materials (e.g., concrete material for earlier stages) or length of the session but also in the pedagogical intentionality that is at the base (what strategy will be used to develop a certain skill and at which level of complexity is desired to promote). (3) Define mindfulness as a latent variable that can be assessed according to the complexity of each developmental period. This implies the use of varied assessment strategies that minimize the difficulties presented by the exclusive use of self-reporting. (4) Address the existing gaps regarding the effects of the MBI on cognitive, socio-emotional, symptomatic, and physiological dependent variables that have not yet been evaluated or have been assessed but cannot be compared. These gaps may be because the research in this field is recent. However, it is important to address them to determine if MBIs effects are consistent or discrepant both within and between developmental periods.

Some limitations of the present review are: (1) Our search included only English and Spanish language peer-reviewed articles. Therefore, this review cannot be claimed as exhaustive, due to the excluded papers. (2) A deep comparison between mindfulness practices in different developmental periods could not be accomplished. Since most authors do not report exercise guidelines, it was not possible to make a more detailed comparison between the contents of each practice (e.g., compare whether the language and way of performing “mindful breathing” are similar or different between periods). (3) Regarding self-report tests, we observed that some of them were applied at different developmental periods. However, we could not compare whether the language was adapted according to each stage of development. (4) We considered only experimental primary studies published in peer-reviewed journals. Nevertheless, it would be interesting to address these limitations in future revisions.

## 5. Conclusions

Mindfulness-based interventions (MBIs) have shown significant effects on child and adolescent populations, particularly in symptom reduction, executive functions, and socioemotional skills. However, these results should be analyzed with caution, considering, the wide variability in terms of types of intervention, assessment strategies, and skills definitions, which makes it practically impossible to compare the effects of MBIs within and between developmental periods. Incorporating a developmental perspective implies defining variables considering latent models, using varied assessment strategies, and incorporating instruments that are comparable to each other, considering the capacities and limitations of each developmental period. This will facilitate an accurate and integrated understanding of the effects of the MBIs in the child and adolescent population within and between developmental stages. The foregoing is essential to understand how MBIs work during childhood and adolescence, regarding what dose, frequency, and type of exercises are the most indicated, and which skills can benefit the most within each developmental stage.

## Figures and Tables

**Table 1:** Systematic Reviews and Meta Analysis search.

ID	Reference	Objective	Screened Data Bases	Search Date	Observed/Declared Limitrations
[45]	Segal, S. C., Vyas, S. S., and Monson, C. M. (2021). A Systematic Review of Mindfulness-Based Interventions in Low-Income Schools. *Mindfulness, 1–16*	Review and summarize the effectiveness of mindfulness-based interventions delivered in low-income schools (Grades 3–9) on psychological functioning.	PsycINFO, Web of Science, PubMed, Scopus, and MEDLINE.	December 2019	Sample: only low-income population, small sample.
[52]	Cilar, L., Štiglic, G., Kmetec, S., Barr, O., and Pajnkihar, M. (2020). Effectiveness of school-based mental well-being interventions among adolescents: A systematic review. *Journal of Advanced Nursing, 76*(8), 2023–2045.	Identify school-based interventions for ensuring mental health and well-being of adolescents, synthesize existing interventions, and summarize the quality of identified studies.	Cochrane Library, PsychARTICLES, Web of Science, CINAHL, and Medline.	March 2019	Sample do not include kids younger than 10 years old. They include non-mindfulness practices.
[51]	Miller, S., Mendelson, T., Lee-Winn, A., Dyer, N. L., and Khalsa, S. B. S. (2020). Systematic review of randomized controlled trials testing the effects of yoga with youth. *Mindfulness, 11*(6), 1336–1353.	Report whether yoga has effects on youth health and mental health outcomes.	PubMed, PsychINFO, Web of Science, Science Citation Index Expanded, Social Sciences Citation Index, Conference Proceedings Citation Index -Science, and Conference Proceedings Citation Index—Social Science and Humanities.	April 2017	Primary studies include yoga practices, not mindfulness
[53]	Emerson, L. M., De Diaz, N. N., Sherwood, A., Waters, A., and Farrell, L. (2020). Mindfulness interventions in schools: Integrity and feasibility of implementation. *International Journal of Behavioral Development, 44*(1), 62–75.	Synthesize the literature on the implementation of school based MBIs and determine the degree to which the interventions align to standards for MBIs	CINAHL, PsycINFO, MEDLINE, EMBASE, Scopus, and Web of Science.	August 2018 and 23 October 2018.	Address the degree to which interventions align to standards.
[54]	McKeering, P. and Y.-S. Hwang (2019). “A systematic review of mindfulness-based school interventions with early adolescents.” *Mindfulness 10*(4): 593–610.	Advance the current understanding of school based MBIs for early adolescents by addressing the identified methodological and empirical limitations of current reviews on school based MBIs.	PsycINFO, ERIC, PsycARTICLES, Education Source, Scopus, Academic OneFile, Medline, PubMed, A+ Education.	October 2017	Sample: only early adolescents.
[36]	Sapthiang, S., Van Gordon, W., and Shonin, E. (2019). Health school-based mindfulness interventions for improving mental health: a systematic review and thematic synthesis of qualitative studies. *Journal of Child and Family Studies, 28*(10), 2650–2658.	Conduct the first systematic review and thematic synthesis to rigorously evaluate the qualitative evidence pertaining to students’ experiences of school based MBIs.	PubMed, Web of Science, Scopus, ProQuest, and Google Scholar.	March 2019	Only qualitative studies.
[1]	Caldwell, D. M., Davies, S. R., Hetrick, S. E., Palmer, J. C., Caro, P., López-López, J. A., ... and Welton, N. J. (2019). School-based interventions to prevent anxiety and depression in children and young people: a systematic review and network meta-analysis. *The Lancet Psychiatry*, 6(12), 1011–1020.	Assess the comparative effectiveness of educational setting-based interventions for preventing depression and anxiety in children and young people.	MEDLINE, Embase, PsycINFO, and Cochrane Central Register of Controlled trials for published and unpublished.	April 2018	Primary outcomes were post-intervention self-report anxiety and depression, wellbeing, suicidal ideation, or self-harm.
[10]	Šouláková, B., Kasal, A., Butzer, B., and Winkler, P. (2019). Meta-review on the effectiveness of classroom-based psychological interventions aimed at improving student mental health and well-being and preventing mental illness. *The Journal of Primary Prevention, 40*(3), 255–278.	Summarize existing evidence from systematic reviews and metanalyses on the effectiveness of school-based psychological interventions aimed at improving student mental health and well-being and preventing mental illness.	PsycINFO, Web of Knowledge, Medline, Embase, and HMIC (Health Management Information Consortium)	No data	These systematic reviews and meta-analyses evaluated the effects of five types of school-based psychological interventions: Mindfulness, Social Emotional Learning, Cognitive Behavioral Therapy, Yoga, and BodyImage.
[35]	Carsley, D., Khoury, B. and Heath, N.L. Effectiveness of Mindfulness Interventions for Mental Health in Schools: a Comprehensive Meta-analysis. *Mindfulness 9*, 693–707 (2018). https://doi.org/10.1007/s12671-017-0839-2	The first objective of this metanalysis is to determine the strength of the effects of school-based mindfulness interventions on mental health and wellbeing outcomes. Subsequently, the second objective is to examine and compare the strength of the effects of the moderators for these interventions based on (1) developmental periods, (2) gender groups, (3) type of mindfulness intervention, and (4) the identity of the facilitator. The current meta-analysis will provide important information on the potential role of individual differences and intervention characteristics across developmental periods in the effectiveness of mindfulness	A systematic review of studies published in PsycINFO, ERIC, Social Work Abstracts, Social Services Abstracts, and CINAHL was conducted.	Data was collected during November 2016 and revised in March 2017.	Sample age: middle childhood, early and late adolescence. They compare strengths of effects and moderators of MBIs, but studies are older than 5 years.
[14]	Burke, C. A. (2010). Mindfulness-based approaches with children and adolescents: a preliminary review of current research in an emergent field. *Journal of Child and Family Studies*, 19(2), 133–144. https://doi.org/10.1007/s10926-009-9282-x.	The aim is to provide a preliminary overview of all the available research in this newly emerging field.	PsychINFO, PSYarticles, BioMed Central, CSA Illumina, Medline, blackwell Synergy, JSTOR, Web of Knowledge Version 4, Science Direct, SpringerLink, Wiley Interscience, and the Cochrane Library, or acquired directly from the author.	No data	Studies are older than 10 years.
[55]	Felver, et al. (2016). “A systematic review of mindfulness-based interventions for youth in school settings.” *Mindfulness 7*(1): 34–45.	The purpose of this paper is to systematically review the current scientific literature base of MBI for youth in school settings summarizing the existing literature and evaluating the methodologies employed in these studies. Specific limitations in the research and recommendations for future studies necessary to advance the nascent field will be identified.	Multiple electronic databases were searched Databases included PsychINFO, ERIC, MEDLINE, and PubMed	Published anytime until June of 2014.	Consider all ages but studies are older than 5 years and do not compare through developmental stages.

**Table 2:** ID reference coding of Primary Articles.

ID	Authors	Year	Title
[57]	Ponsoda, F.C.	2017	The effect of an out-of-school mindfulness program on adolescents’ stress reduction and emotional wellbeing
[58]	Devcich, D.A.; Bernay, R.; Graham, E.	2017	Effectiveness of a Mindfulness-Based Program on School Children’s Self-Reported Well-Being: A Pilot Study Comparing Effects with An Emotional Literacy Program
[59]	Quach, D.; Mano, Gibler, R.C.; Jastrowski Mano, K.E.	2017	Does Home Practice Compliance Make a Difference in the Effectiveness of Mindfulness Interventions for Adolescents?
[60]	Tarrasch, R; Margalit-Shalom, L; Berger, R.	2017	Enhancing Visual Perception and Motor Accuracy among School Children through a Mindfulness and Compassion Program
[61]	Berger, R; Brenick, A.; Tarrasch, R.	2018	Reducing Israeli-Jewish Pupils’ Outgroup Prejudice with a Mindfulness and Compassion-Based Social-Emotional Program
[62]	Lemberger–Truelove, M.E.; Carbonneau, K. J., Atencio, D. J.; Zieher, A. K.; Palacios, A. F.	2018	Self-Regulatory Growth Effects for Young Children Participating in a Combined Social and Emotional Learning and Mindfulness-Based Intervention
[63]	Modi, S.; Joshi, U; Narayanakurup, D.	2018	To what extent is mindfulness training effective in enhancing self-esteem, self-regulation and psychological well-being of school going early adolescents?
[64]	Rodriguez-Ledo, C; Orejudo, S., Cardoso, M. J., Balaguer, Á., & Zarza-Alzugaray, J	2018	Emotional Intelligence and Mindfulness: Relation and Enhancement in the Classroom with Adolescents
[65]	Tarrasch, R.	2018	The Effects of Mindfulness Practice on Attentional Functions Among Primary School Children
[66]	Viglas, M. & Perlman, M.	2018	Effects of a Mindfulness-Based Program on Young Children’s Self-Regulation, Prosocial Behavior and Hyperactivity
[67]	Wood, L; Roach, A. T., Kearney, M. A., & Zabek, F.	2018	Enhancing executive function skills in preschoolers through a mindfulness-based intervention: A randomized, controlled pilot study
[68]	Zelazo, P. D.; Forston, J. L.; Masten, A. S. & Carlson, S. M.	2018	Mindfulness Plus Reflection Training: Effects on Executive Function in Early Childhood
[69]	Bauer, C. C; Caballero, C.; Scherer, E; West, M. R.; Mrazek, M. D.; Phillips, D. T.; Whitfield–Gabrieli, S; Gabrieli, J. D.	2019	Mindfulness Training Reduces Stress and Amygdala Reactivity to Fearful Faces in Middle-School Children
[70]	Janz, P; Dawe, S; Wyllie, M.	2019	Mindfulness-Based Program Embedded Within the Existing Curriculum Improves Executive Functioning and Behavior in Young Children: A Waitlist Controlled Trial
[71]	Rawlett, K. E.; Friedmann, E; Thomas, S. A.	2019	Mindfulness-based intervention with an attentional comparison group in at risk young adolescents: a pilot randomized controlled trial
[72]	Alampay, L. P..; Galvez Tan, L. J. T., Tuliao, A. P., Baranek, P., Ofreneo, M. A., Lopez, G. D., ... & Guintu, V.	2020	A Pilot Randomized Controlled Trial of a Mindfulness Program for Filipino Children
[73]	Berti, S. & Cigala, A.	2020	Mindfulness for Preschoolers: Effects on Prosocial Behavior, Self-Regulation and Perspective Taking
[74]	Franco, C.; Soriano, E; Amutio, A. & Mañas, I.	2020	Improving motivation in Latin American immigrants through a mindfulness-based program: A randomized study
[75]	Volanen, S. M; Lassander, M; Hankonen, N; Santalahti, P; Hintsanen, M; Simonsen, N; Raevuori, A; Mullola, S; Vahlberg, T; But, A; Suominen, S	2020	Healthy learning mind—Effectiveness of a mindfulness program on mental health compared to a relaxation program and teaching as usual in schools: A cluster-randomized controlled trial
[76]	Ghiroldi, S.; Scafuto, F.; Montecucco, N. F.; Presaghi, F. & Iani, L.	2020	Effectiveness of a School-Based Mindfulness Intervention on Children’s Internalizing and Externalizing Problems: the Gaia Project
[77]	Güldal, Ş. & Satan, A.	2020	The effect of mindfulness-based psychoeducation program on adolescents’ character strengths, mindfulness, and academic achievement
[78]	Kim, E; Jackman, M. M.; Jo, S. H.; Oh, J.; Ko, S. Y.; McPherson, C. L.; Hwang, Y. S.; Singh, N. N.	2020	Effectiveness of the Mindfulness-Based Open Mind-Korea (OM-K) Preschool Program
[79]	Lahtinen, O; Salmivalli, C.	2020	An Effectiveness Study of a Digital Mindfulness-Based Program for Upper Secondary Education Students
[80]	Lam, K. & Seiden, D.	2020	Effects of a Brief Mindfulness Curriculum on Self-reported Executive Functioning and Emotion Regulation in Hong Kong Adolescents
[81]	Lassander, M.; Hintsanen, M.; Suominen, S.; Mullola, S.; Fagerlund, Å; Vahlberg, T.; Volanen, S. M.	2020	The Effects of School-based Mindfulness Intervention on Executive Functioning in a Cluster Randomized Controlled Trial
[82]	Pinazo, D.; García-Prieto, L. T.; García–Castellar, R.	2020	Implementation of a program based on mindfulness for the reduction of aggressiveness in the classroom
[83]	Frank, J. L. B; Broderick, P. C.; Oh, Y.; Mitra, J.; Kohler, K.; Schussler, D. L.; Geier, C.; Roeser, R. W.; Berrena, E.; Mahfouz, J.; Levitan, J.; Greenberg, M. T.	2021	The Effectiveness of a Teacher-Delivered Mindfulness-Based Curriculum on Adolescent Social-Emotional and Executive Functioning

## Data Availability

Not applicable.
